# A lipid metabolism and lysosome-based risk signature for prognosis and immune response prediction in uterine corpus endometrial carcinoma

**DOI:** 10.3389/fgene.2025.1594682

**Published:** 2025-09-08

**Authors:** Yuanyuan Zhu, Pusheng Yang, Shu Zhang

**Affiliations:** ^1^ Shanghai Key Laboratory of Gynecology Oncology, Department of Gynecology and Obstetrics, Renji Hospital, Shanghai Jiao Tong University School of Medicine, Shanghai, China; ^2^ Shanghai Key Laboratory of Maternal Fetal Medicine, Shanghai Institute of Maternal-Fetal Medicine and Gynecologic Oncology, Shanghai First Maternity and Infant Hospital, Tongji University School of Medicine, Shanghai, China

**Keywords:** lipid metabolism, lysosome, prognostic signature, immunotherapy, uterine corpus endometrial carcinoma

## Abstract

**Background:**

The dysregulation of genes related to lipid metabolism and lysosomal function has been reported to significantly contribute to tumor progression. In this study, we systematically explored the roles played by lipid metabolism and lysosomes in uterine corpus endometrial carcinoma (UCEC), aiming to identify potential biomarkers for predicting prognosis and immune checkpoint therapy efficacy.

**Methods:**

Genes associated with lipid metabolism and lysosomal function were retrieved from the MSigDB and GO databases. Transcriptomic data and clinical information of patients were acquired from The Cancer Genome Atlas database. A prognostic model was constructed using consensus clustering, univariate Cox regression, and LASSO regression. ROC curves, Kaplan-Meier plots, and calibration curves were employed to assess the predictive capacity of the model, while ssGSEA, TIDE, and IPS were used to evaluate the response of high- and low-risk groups to immunotherapy. Drug sensitivity was assessed with the “oncoPredict” R package. Given that we identified a strong association between *PLAAT1* and CD8^+^ T-cell infiltration, this gene was selected for loss-of-function assays in UCEC cells, including the evaluation of their proliferative, invasive, and migratory potential.

**Results:**

An eight-gene (*LAMP3*, *RNF183*, *EEF1A2*, *PLAAT1*, *ELAPOR1*, *B4GALT1*, *ATP10B*, and *PLA2G10*) risk signature based on lipid metabolism and lysosomal function was constructed to distinguish high-risk and low-risk UCEC patients. Subsequent analyses showed that patients classified as high risk had higher TIDE scores, whereas those categorized as low risk exhibited higher MSI scores and greater levels of CD8^+^ T-cell infiltration. All evidence suggested that patients in the low-risk group displayed greater immunogenicity and sensitivity to both immunotherapy and chemotherapy. Analysis using the TIMER database indicated that among the eight risk genes, *PLAAT1* showed the strongest association with CD8^+^ T-cell immune infiltration in UCEC. Cytological experiments confirmed that the knockdown of *PLAAT1* effectively suppressed the proliferation and motility of endometrial cancer cells.

**Conclusion:**

We constructed a risk prognostic model for UCEC based on a combination of lysosomal- and lipid metabolism-related genes. Our findings highlight the oncogenic potential of PLAAT1 in endometrial cancer and provide novel insights into the diagnosis and therapy of this cancer type.

## 1 Introduction

Endometrial carcinoma is one of the most common gynecological malignancies worldwide. In the United States, there are expected to be approximately 67,880 new cases of endometrial cancer in 2024 (Siegel et al., 2024). Globally, the age-standardized incidence and mortality rates of endometrial cancer significantly increased by 0.69% per year between 1990 and 2019 (Gu et al., 2021). The incidence rate of endometrial cancer has increased in many countries over recent decades, largely attributable to changes in dietary lifestyles and the escalating prevalence of obesity (Sung et al., 2021). While patients diagnosed at an early stage have a relatively favorable prognosis, those diagnosed at an advanced stage or presenting with recurrent disease display adverse prognostic outcomes (Pirš et al., 2022; NIH, 2023). Despite the continual advancements in therapies targeted at patients with advanced or recurrent disease, the efficacy of these treatments varies widely due to the heterogeneity of endometrial cancer (Tung et al., 2022; Ott et al., 2017; Marabelle et al., 2020). Consequently, a critical priority is the identification of suitable molecular markers and the customization of personalized treatments to ameliorate the prognosis of patients with endometrial carcinoma.

Obesity is increasingly recognized as a pivotal contributor to the pathogenesis of endometrial cancer (Raglan et al., 2019; Shaw et al., 2016) and is inextricably linked to abnormal lipid metabolism. Tan et al. (2022) built a prognostic risk signature for uterine corpus endometrial carcinoma (UCEC), encompassing 11 lipid metabolism-related genes. Research has demonstrated that the dysregulation of enzymes and signaling molecules involved in lipid metabolism directly or indirectly influences oncogenic processes (Kim et al., 2023). Emerging evidence has indicated that lysosomes also have an essential function in lipid metabolism (Fröhlich and González Montoro, 2023; Shin et al., 2022; Settembre et al., 2013). While lysosomes were initially identified as static organelles involved in the degradation and recycling of cellular waste, recent work has demonstrated that they also play a role in the regulation of lipid homeostasis and cancer progression (de Duve, 2005). For instance, the growth of hepatocellular carcinoma (HCC) cells was inhibited by the BNIP3-mediated facilitation of lipid droplet turnover in lysosomes (Berardi et al., 2022). Lysosomal lipid switching can influence nutrient signaling through mTORC1, ultimately impacting cell growth and metabolism (Wallroth et al., 2019; De Santis et al., 2023). The storage and release of cholesterol in lysosomes can also modulate cancer cell behavior (Bartel et al., 2017; Nguyen et al., 2022). Combined, these observations suggest that intracellular lipid metabolism and lysosomes are strongly linked. However, the prognostic significance of lysosome-associated genes in UCEC prognosis remains unclear, as does the putative interplay between lipid metabolism and lysosomes in this cancer type.

In this study, our objective was to construct a risk prognostic model based on lipid metabolism- and lysosome-associated genes and to evaluate the diagnostic efficacy of this model. We identified eight key diagnostic biomarkers *(LAMP3, RNF183, EEF1A2, PLAAT1, ELAPOR1, B4GALT1, ATP10B, and PLA2G10)* and clarified whether these biomarkers can guide immune checkpoint inhibitor therapy and chemotherapy for UCEC patients. Given that we identified a strong association between PLAAT1 and CD8^+^ T-cell infiltration, we then used *in vitro* experiments to clarify the impact of PLAAT1 on the biological behaviour of endometrial cancer cells. These findings may contribute to providing more specific treatments for UCEC patients to improve their prognosis.

## 2 Methods

### 2.1 Data acquisition and processing

The R package “TCGAbiolinks” was used to download RNA-seq data (TPM and Counts values) and clinical data (comprising 554 cancer tissue samples and 35 healthy adjacent tissue samples) from The Cancer Genome Atlas (TCGA) dataset for UCEC patients (Colaprico et al., 2016). The inclusion criteria for UCEC patients, along with clinical information for the eligible patients, is listed in Supplementary Table S1. A total of 1,407 genes related to lipid metabolism (LMRGs) were downloaded from the Molecular Signatures Database (MSigDB) (http://www.gsea-msigdb.org/) (Supplementary Table S2), and 893 lysosome-relevant genes (LYRGs) were retrieved from the Gene Ontology (GO) (https://geneontology.org/) and MSigDB databases (Supplementary Table S3).

### 2.2 Identification of prognosis-associated differentially expressed lipid metabolism-related genes

Using a |log2FC| > 1 and *padj*. < 0.01 as criteria, 5,507 TCGA-DEGs between UCEC and normal tissues were screened *via* the “DESeq2” R package (Love et al., 2014). Of these TCGA-DEGs, 418 were identified as differentially expressed LMRGs (DE-LMRGs). The effect of the 418 DE-LMRGs on UCEC prognosis was then assessed by Kaplan-Meier survival analysis using the “survival” and “survminer” R packages. Finally, 57 putative prognosis-related genes (PRGs) were selected for further investigation.

### 2.3 Unsupervised clustering for prognosis-related genes (PRGs)

Based on the expression profiles of the 57 putative PRGs, UCEC patients were classified into distinct molecular subgroups *via* unsupervised clustering. To ensure the stability of the clustering results, the analysis was repeated 1,000 times using the “ConsensusClusterPlus” package (Wilkerson and Hayes, 2010). Additionally, principal component analysis (PCA) was performed using the “stats” R package to validate and visualize the clustering results.

### 2.4 Risk model construction

Differential expression analysis was conducted using the “DESeq2″ package to identify genes exhibiting differential expression among the three lipid metabolism subtypes (LM-DEGs). Genes with *padj*. values of less than 0.01 and an absolute log2 fold change greater than 1 were considered significantly differentially expressed. The intersection of the 5,507 TCGA-DEGs, 893 LYRGs, and 1,085 LM-DEGs yielded 26 overlapping genes, which were selected for subsequent analyses. Univariate Cox regression analysis was performed on the 26 genes, leading to the identification of 11 genes with prognostic potential. Following least absolute shrinkage and selection operator (LASSO) regression analysis of the 11 candidate genes, the eight exhibiting the strongest association with UCEC prognosis were selected and were subsequently used to construct a prognostic model for UCEC.

### 2.5 Performance assessment and functional enrichment analysis

To evaluate the prognostic capacity of the model in UCEC, the patients were initially ranked into low- and high-risk groups based on their calculated risk scores. Subsequently, potential disparities in survival outcomes between the two groups were investigated. Several analytical approaches were employed to assess the accuracy of the model, including K-M survival, ROC curve, and Cox regression analyses. Additionally, to assess the reliability of the model and thereby enhance its clinical applicability, a nomogram and a calibration curve were created. Gene Ontology (GO) term and Kyoto Encyclopedia of Genes and Genomes (KEGG) pathway enrichment analyses were conducted employing the KOBAS web server (http://kobas.cbi.pku.edu.cn) (Bu et al., 2021). Gene Set Variation Analysis (GSVA) was performed to identify sets of enriched genes among all samples within the UCEC cohort (Hänzelmann et al., 2013).

### 2.6 Tumor mutational burden (TMB) and immune landscape analyses

The R package “maftools” was used to compute the TMB for each patient, and the Spearman correlation coefficient between the risk score and the TMB was subsequently calculated (Mayakonda et al., 2018). The single-sample Gene Set Enrichment Analysis (ssGSEA) algorithm was employed to quantify immune cell infiltration using the “GSVA” package in R. The Tumor Immune Dysfunction and Exclusion (TIDE) score was used to estimate tumor immune escape (Jiang et al., 2018). The ability of the developed risk features to predict anticancer treatment efficacy was validated using Immunophenoscore (IPS) data and the “oncoPredict” software package (Charoentong et al., 2017; Maeser et al., 2021).

### 2.7 Cell culture

The origin and culture methods of the four endometrial cancer cell lines used in this study (Ishikawa, ECC-1, HEC-1A, and HEC-1B) were as previously described (Yang et al., 2022). Human phospholipase A and acyltransferase 1 (*PLAAT1*)-specific siRNA and control siRNA were sourced from Gene Pharma (Shanghai, China). The sequences used for the silencing of *PLAAT1* are presented in Supplementary Table S4.

### 2.8 Quantitative real-time PCR and western blot

Total RNA was extracted from logarithmically growing cells with Trizol reagent (Invitrogen) according to the manufacturer’s instructions. Total RNA (500 ng) was reverse transcribed into cDNA using PrimeScript RT Master Mix (ES Science, Shanghai, China). qPCR was performed with the primers listed in Supplementary Table S5 and SYBR Green Master Mix (ES Science). Proteins were separated by SDS-PAGE electrophoresis, initially at 80 V until the samples migrated through the stacking gel, followed by 120 V until the target protein markers were adequately resolved. The proteins were then transferred to a PVDF membrane at a constant current of 250 mA for 40 min. The membrane was blocked with 5% skim milk for 1 h, incubated with primary antibody (PLAAT1, 1:500 dilution) at 4°C overnight, and subsequently probed with a secondary antibody (1:5,000 dilution) at room temperature for 90 min, followed by TBST washes. Finally, the membrane was incubated with ECL substrate for 1–2 min and visualized using a chemiluminescence imaging system. The detailed information of the antibodies used is provided below: anti-ACTB (81115-1-RR, Proteintech, China), anti-PLAAT1 (YN3890, Immunoway, China).

### 2.9 Cell proliferation assay

To compare the proliferative capacity of endometrial cancer cells after different treatments, a CCK-8 assay (SB-CCK8S, Share-Bio, China) was performed following the manufacturer’s instructions. HEC-1A cells (3,000 per well) were seeded in a 96-well plate, and the absorbance at 450 nm was measured initially after cell adhesion, and then every 24 h for a total of 5 times. In the colony formation assay, cells were seeded in a 24-well plate at a density of 500 cells per well. After 12 days of incubation, the colonies were stained and imaged for subsequent analysis.

### 2.10 Cell migration and invasion assay

Cell suspensions (4 × 10^4^ HEC-1A cells) were added to the upper chambers (8-μm pore; coated or not with Matrigel) of 24-well Transwell plates for cell migration and invasion assays. The invasion assay was conducted for a total of 48 h, while the migration assay was conducted for a total of 36 h. After fixation and staining, the cells were imaged and counted.

### 2.11 Statistical analysis

Statistical analyses were conducted using R software (v.4.1.3) and GraphPad Prism 9.0. The presented results are based on a minimum of three independent experiments and are expressed as means ± SD. Statistical significance was determined by comparing *p*-values, with a threshold of <0.05 considered significant.*p < 0.05, **p < 0.01, ***p < 0.001, ****p < 0.0001, ns: not significant.

## 3 Results

### 3.1 Identification and clustering of LMRGs

**FIGURE 1:**
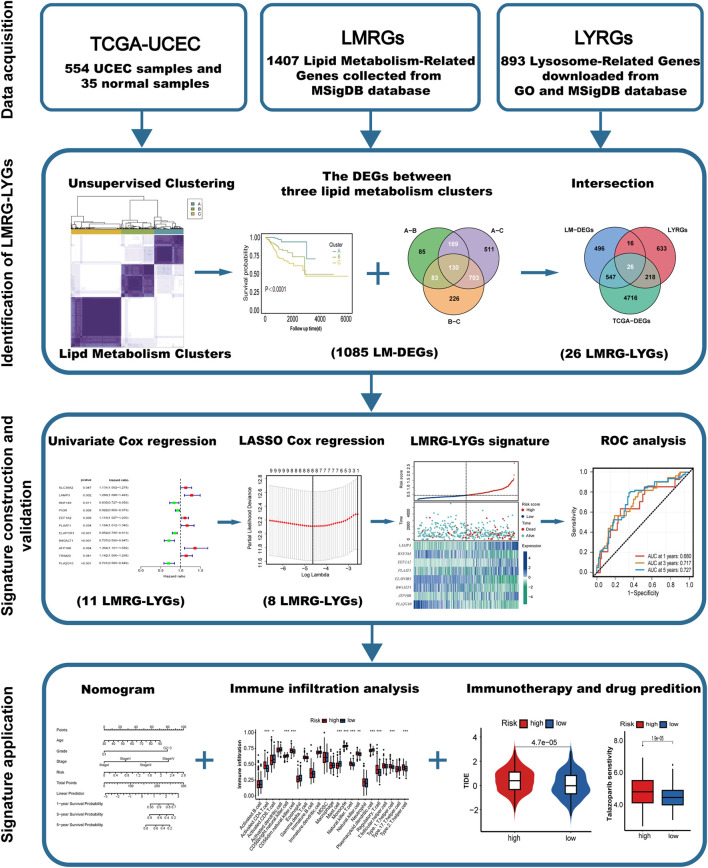
Study flowchart.

**FIGURE 2:**
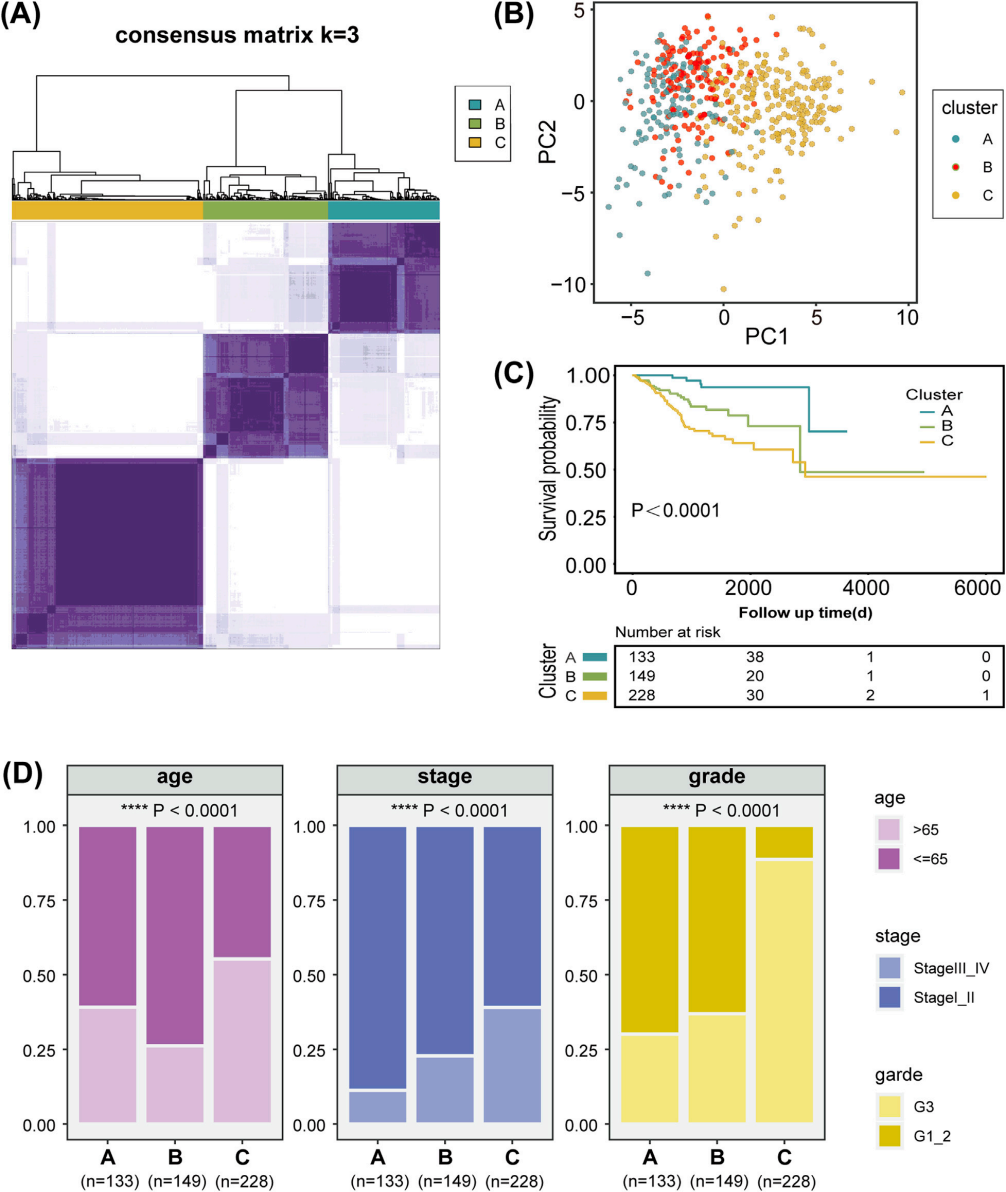
Characterization of lipid metabolism patterns in uterine corpus endometrial carcinoma (UCEC). **(A)** Consensus clustering of 57 prognosis-related genes (PRGs) in the TCGA-UCEC cohort. **(B)** Principal component analysis of three lipid metabolism clusters. **(C)** Kaplan-Meier analysis of the three lipid metabolism clusters. **(D)** Analysis of the constituent ratio of clinical characteristics in the different lipid metabolism clusters.

The overall workflow of the study is illustrated in Figure 1. We retrieved 1,407 LMRGs from the GSEA website and identified 418 DE-LMRGs in UCEC patients from TCGA cohort. A batch survival analysis of these 418 DE-LMRGs revealed that 57 of them were significantly associated with overall survival (OS). A consensus cluster consisting of these 57 genes was constructed with the “ConsensusClusterPlus” package. The results showed that TCGA-UCEC cohort was effectively stratified into three clusters—cluster A, with 133 samples; cluster B, with 149; and cluster C, with 228 samples (Figure 2A; Supplementary Figure S1A, B). A PCA plot of the three subgroups is illustrated in Figure 2B. Subsequent survival analysis uncovered notable disparities among the three clusters, with cluster A exhibiting a particularly notable survival advantage (Figure 2C). We also investigated the relationship between the subgroups and clinical-pathological parameters in UCEC patients (Figure 2D). Cluster A had the lowest proportion of G3 and stage III–IV patients, followed by cluster B, and then cluster C. The expression patterns of the 57 genes used for clustering are shown as a heatmap in Supplementary Figure S1C.

### 3.2 Construction and validation of a LMRG/LYG-based signature

**FIGURE 3:**
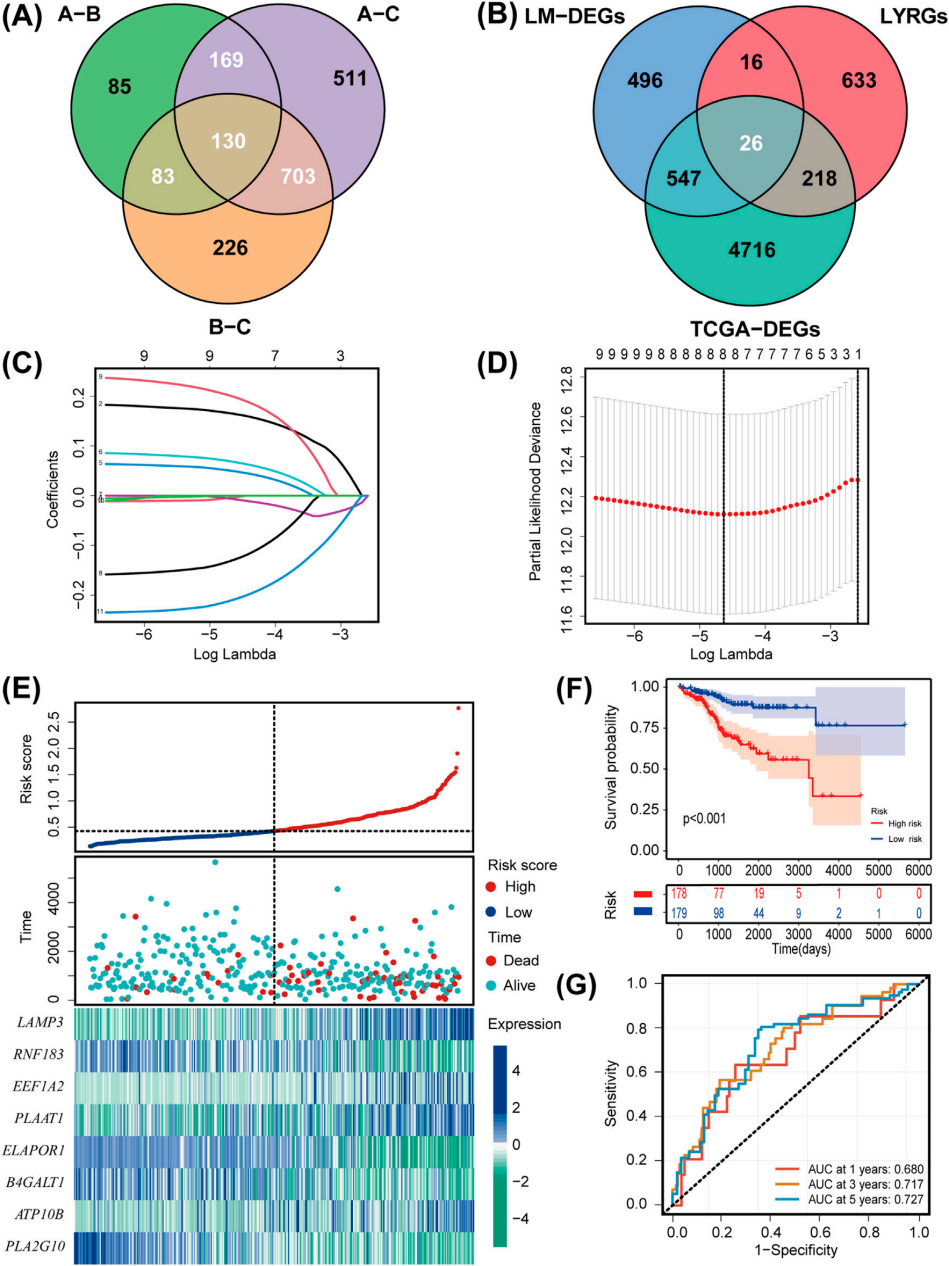
Establishment and evaluation of the LMRG/LYG-based signature. **(A)** A Venn diagram of the 1,085 LM-DEGs in the different comparison groups (A vs. B, A vs. C, and B vs. C). **(B)** A Venn diagram of the LMRG-LYGs among the 5,507 TCGA-DEGs, 893 LYRGs, and 1,085 LM-DEGs. **(C,D)** The LASSO regression analysis identified eight risk genes associated with uterine corpus endometrial carcinoma (UCEC) prognosis. **(E)** The expression of the eight risk genes in the low-risk and high-risk groups in the training set and the distribution of survival outcomes within these groups. **(F)** Survival curve comparison of patients in the different risk groups in the training set. **(G)** ROC curves from the training set, demonstrating the robustness of the prognostic model.

We identified a total of 1,085 DEGs between the three lipid metabolism subgroups (Figure 3A). As illustrated in the Venn diagram in Figure 3B, the intersection of TCGA-DEGs, LYRGs, and LM-DEGs yielded a total of 26 overlapping genes, defined as LMRG-LYGs. To construct the UCEC prognostic model, we further screened the LMRG-LYGs using univariate Cox regression analysis, which revealed that 11 genes were significantly associated with UCEC prognosis (Supplementary Figure S2A). Subsequently, employing LASSO Cox regression analysis based on these 11 candidate genes, we developed a prognostic model consisting of eight genes (Figures 3C,D). The risk score was calculated based on these eight genes using the following formula: risk score = [expression (LAMP3) × (0.16454)] + [expression (RNF183) × (−0.00337)] + [expression (EEF1A2) × (0.04929)] + [expression (PLAAT1) × (0.06792)] + [expression (ELAPOR1) × (−0.00696)] + [expression (B4GALT1) × (−0.12904)] + [expression (ATP10B) × (0.19877)] + [expression (PLA2G10) × (−0.20899)]. Additionally, gene expression analyses showed that all eight risk genes were upregulated in tumor samples compared to normal samples (Supplementary Figure S2B). Meanwhile, we used immunohistochemistry data from The Human Protein Atlas database to confirm the protein levels of these eight risk genes (Supplementary Figure S3), and we presented the eight risk genes’ biological functions and annotations (Supplementary Table S6). To understand the prognostic value of these genes, survival curves were plotted (Supplementary Figure S2C). The training set was divided into low-risk and high-risk UCEC groups based on the median risk score, with the latter exhibiting markedly higher mortality than the former. The differential expression of the eight genes between the two groups is depicted in a heatmap in Figure 3E. Subsequent Kaplan-Meier survival curve analysis revealed that a high-risk score was correlated with an unfavorable prognosis (Figure 3F). ROC plots were generated to evaluate the diagnostic performance of the model, yielding area under the curve (AUC) values of 0.680 (1-year OS), 0.717 (3-year OS), and 0.727 (5-year OS) (Figure 3G). The prognostic model was tested in both the UCEC testing set and the entire UCEC cohort, consistently confirming the robust predictive value of the LMRG/LYG-based signature (Supplementary Figure S2D–G).

### 3.3 Integrated analysis of LMRG/LYG-based signature

**FIGURE 4:**
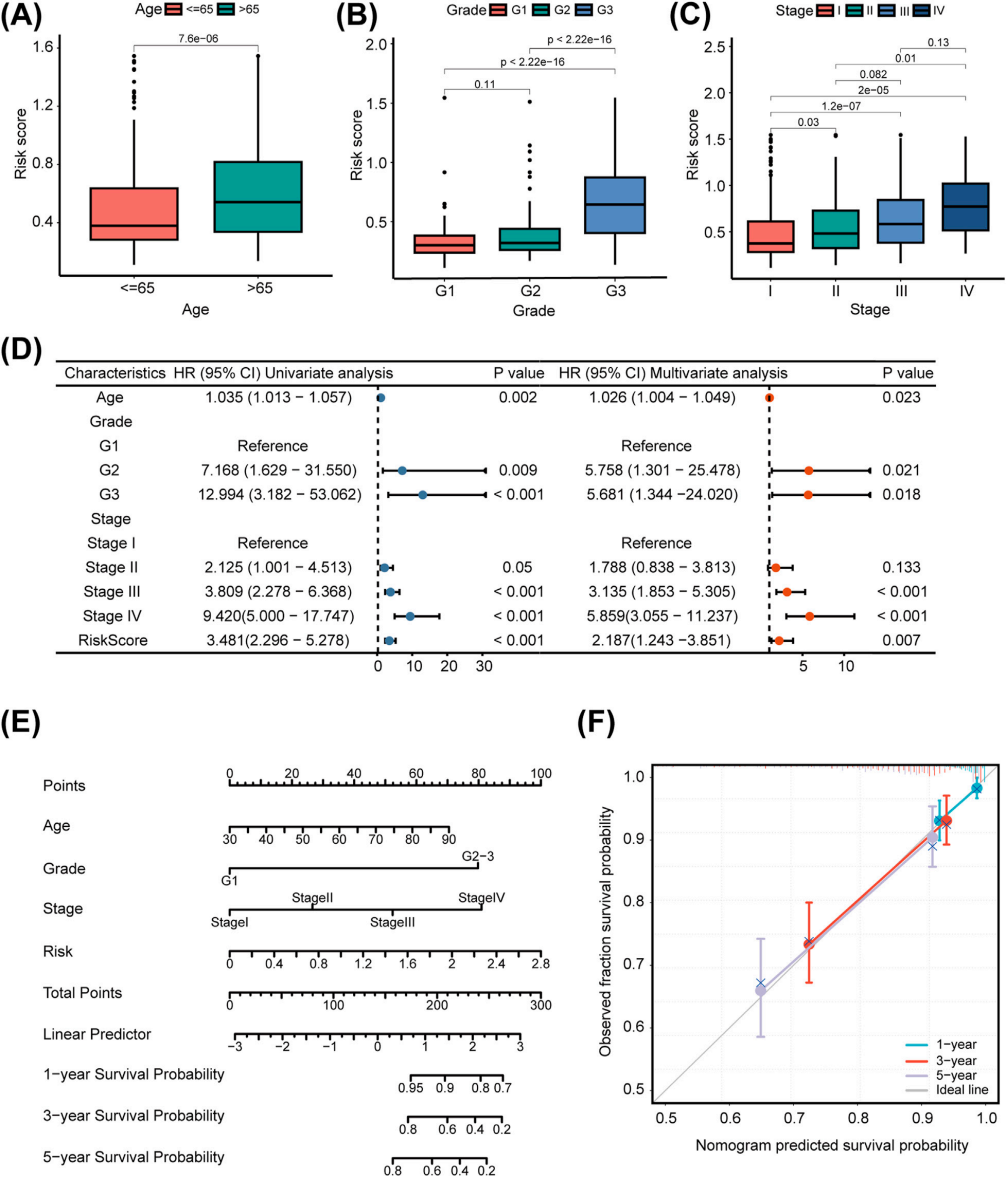
The clinical applicability of the risk model. The association of the risk score with uterine corpus endometrial carcinoma (UCEC) patients’ age **(A)**, tumor grade **(B)**, and tumor stage **(C)**. **(D)** Univariate and multivariate Cox regression analysis of clinicopathological factors and the risk score. **(E)** Nomogram for predicting prognosis in uterine corpus endometrial carcinoma (UCEC) patients. **(F)** Calibration curve of the nomogram.

To comprehensively evaluate the clinical applicability of our LMRG/LYG-based signature, box plots were used to illustrate the correlation between risk scores and various clinicopathological features. We observed that higher risk scores were associated with older age, advanced histologic grade, and advanced clinical stage (Figures 4A–C). Further analysis using a forest plot identified significant independent associations between clinical characteristics (age, grade, and stage) and the OS of UCEC patients. Moreover, the risk score itself, in addition to being associated with these clinical features, also served as an independent prognostic factor, underscoring the high accuracy of our features (Figure 4D). To determine the probability of OS (1-, 3-, and 5-year) for each patient individually, a nomogram was established by integrating clinicopathological features and risk scores (Figure 4E). Meanwhile, the calibration curves for 1-, 3-, and 5-year survival demonstrated that there was strong agreement between the survival rates predicted by the nomogram and the observed survival rates (Figure 4F). This suggested that our nomogram could accurately predict the prognosis of UCEC patients.

### 3.4 Association of the LMRG/LYG-based signature with tumor genomic profiles

**FIGURE 5:**
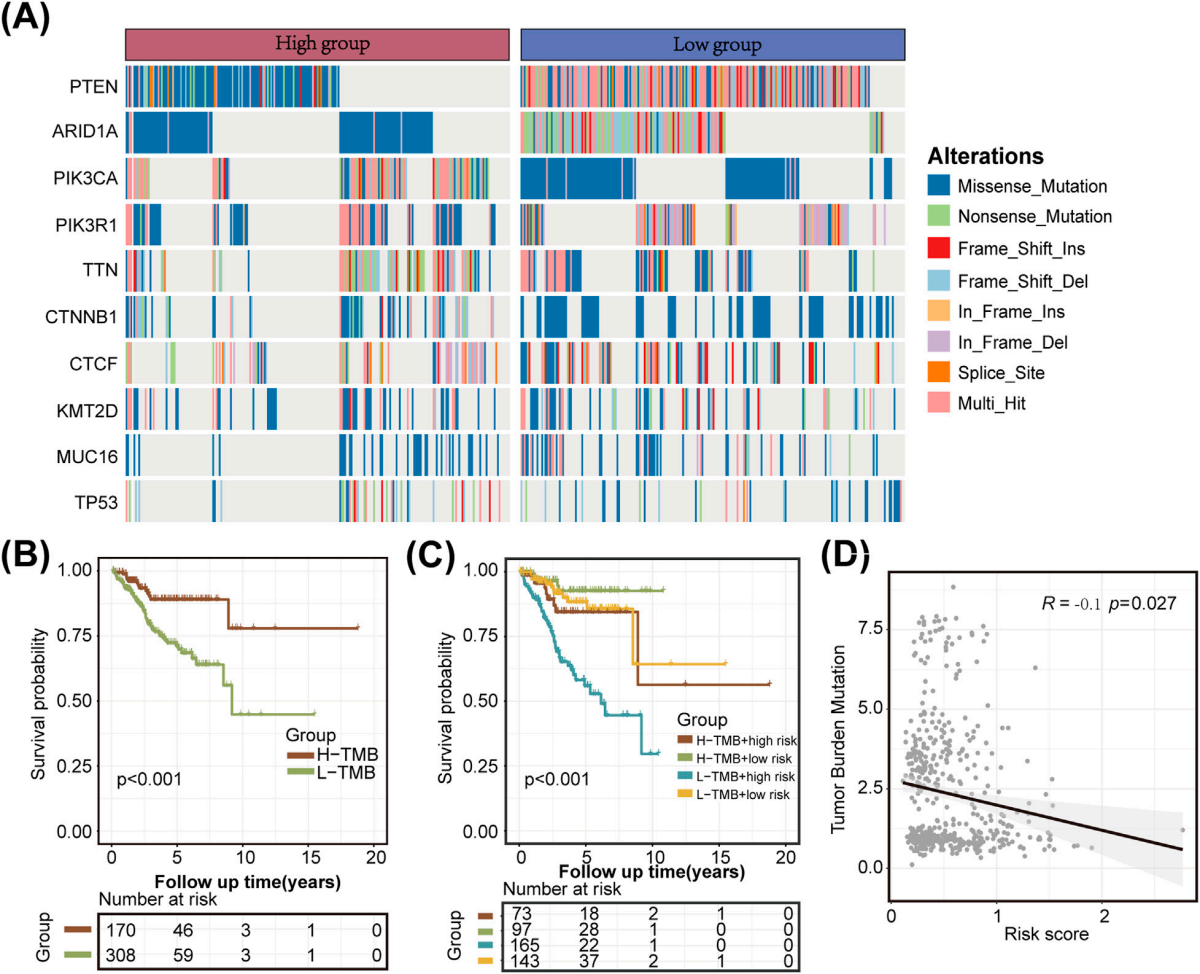
Tumor mutation analysis. **(A)** A waterfall plot illustrating the mutational differences of the 10 genes with the highest mutation rates between the two risk groups identified using the LMRG/LYG-based signature. **(B)** Kaplan-Meier survival curves for the tumor mutational burden (TMB) subgroups. **(C)** Kaplan-Meier survival curves for the combined effects of risk score and TMB. **(D)** A scatter plot of the correlation between the TMB and the risk score.

The TMB can greatly influence anticancer immunity, implying that it holds predictive value for both clinical responses to immune checkpoint inhibitor therapies and the prognosis of UCEC patients. To further evaluate the predictive ability of our constructed LMRG/LYG-based signature and elucidate the tumor mutation landscape in the different risk groups, we first identified the 10 genes exhibiting the highest mutation frequency (Figure 5A). The low-risk group demonstrated a significantly higher prevalence of mutations in most of these genes compared to the high-risk group, and subsequent survival analysis revealed a strong association between a high TMB and improved OS (Figure 5B). To explore the synergistic effect of the TMB and risk score on outcome prediction in more detail, we divided patients into subgroups based on both metrics and observed notable statistical differences in survival rates across the different groups. The cohort with a high TMB in combination with a low risk score had the highest OS rate, while patients with a low TMB and a high risk score demonstrated the lowest OS rate (Figure 5C). Overall, our analysis showed that the risk score was negatively correlated with the TMB, suggesting that the former can serve as a reliable indicator of the latter (Figure 5D). These findings underscore the potential of the LMRG/LYG-based risk score as a predictive tool for responses to immune checkpoint inhibitor (ICI) therapy.

### 3.5 Functional enrichment analysis of the DEGs between the two risk groups

**FIGURE 6:**
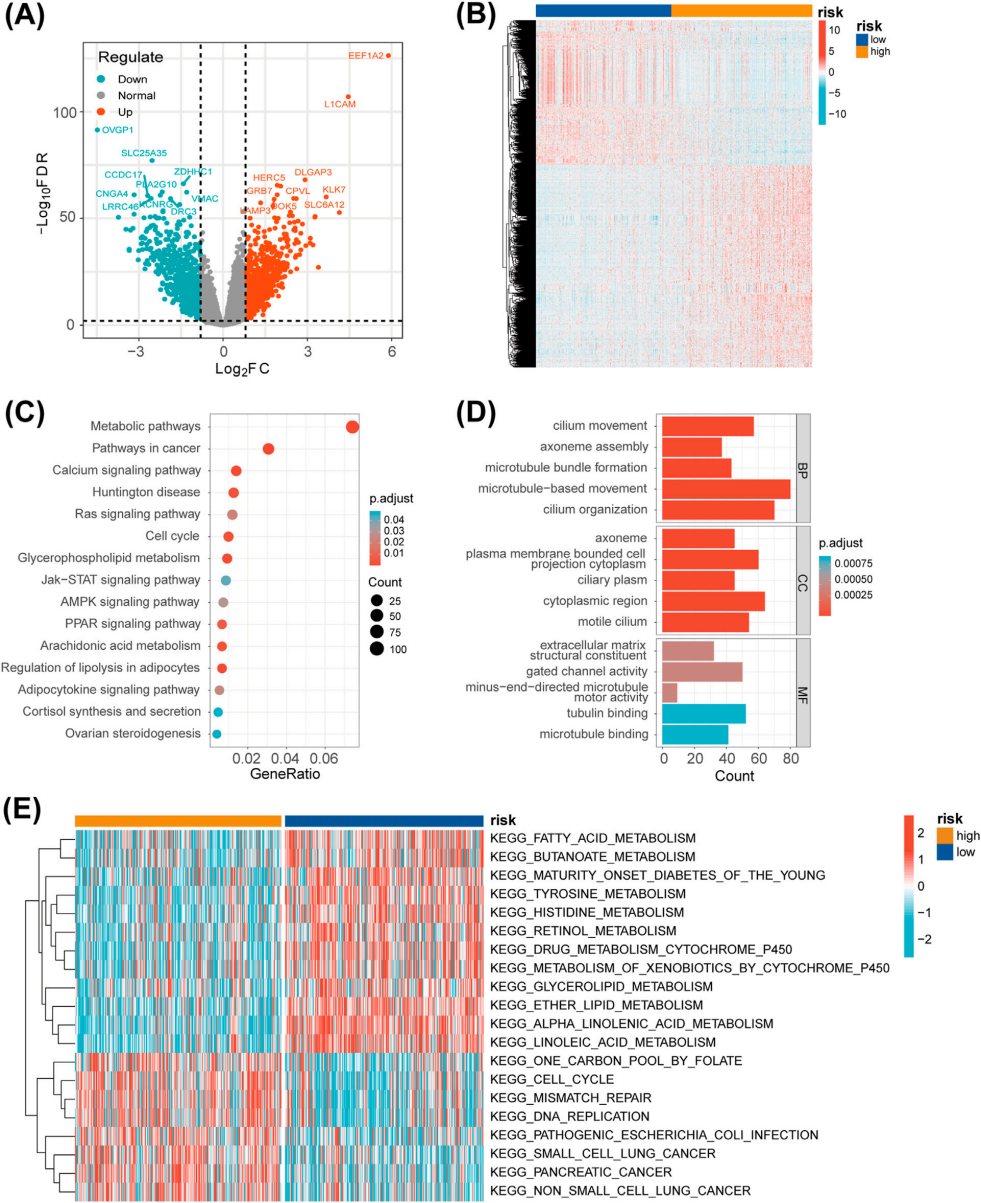
Functional enrichment analysis. **(A)** A volcano plot of the differentially expressed genes (DEGs) between the two risk groups. **(B)** A heatmap showing the expression levels of the DEGs between the two risk groups. **(C)** A bubble chart displaying the results of the KEGG analysis, which clarify the biological processes behind the LMRG/LYG-based signature. **(D)** A bar chart of the GO analysis results, which clarify the biological processes behind the LMRG/LYG-based signature. **(E)** Heat map of the 20 pathways identified by GSVA; blue represents downregulated pathways and red represents activated pathways.

Applying the criteria of *padj*. < 0.01 and |log2FC| > 0.8, we identified 1,514 DEGs between the high-risk and low-risk groups. These DEGs were visualized using a volcano plot and a heatmap (Figures 6A,B). To better understand the significance of the LMRG/LYG-based signature, KEGG and GO enrichment analyses were performed based on the DEGs between the two risk groups. KEGG analysis identified significant enrichment in several metabolic pathways, including Arachidonic acid metabolism, Cortisol synthesis and secretion, and Glycerophospholipid metabolism (Figure 6C; Supplementary Table S7). Moreover, GO analysis results indicated that these DEGs were significantly associated with terms such as microtubule bundle formation, extracellular matrix, and gated channel activity (Figure 6D; Supplementary Table S8). Furthermore, through GSVA, we identified gene enrichment in pathways such as DNA replication, cell cycle, and mismatch repair in the high-risk group. In contrast, the low-risk group exhibited enrichment of genes linked to pathways such as fatty acid metabolism, glycerolipid metabolism, and linoleic acid metabolism (Figure 6E; Supplementary Table S9).

### 3.6 Predicting the sensitivity of patients to antitumor therapy

**FIGURE 7:**
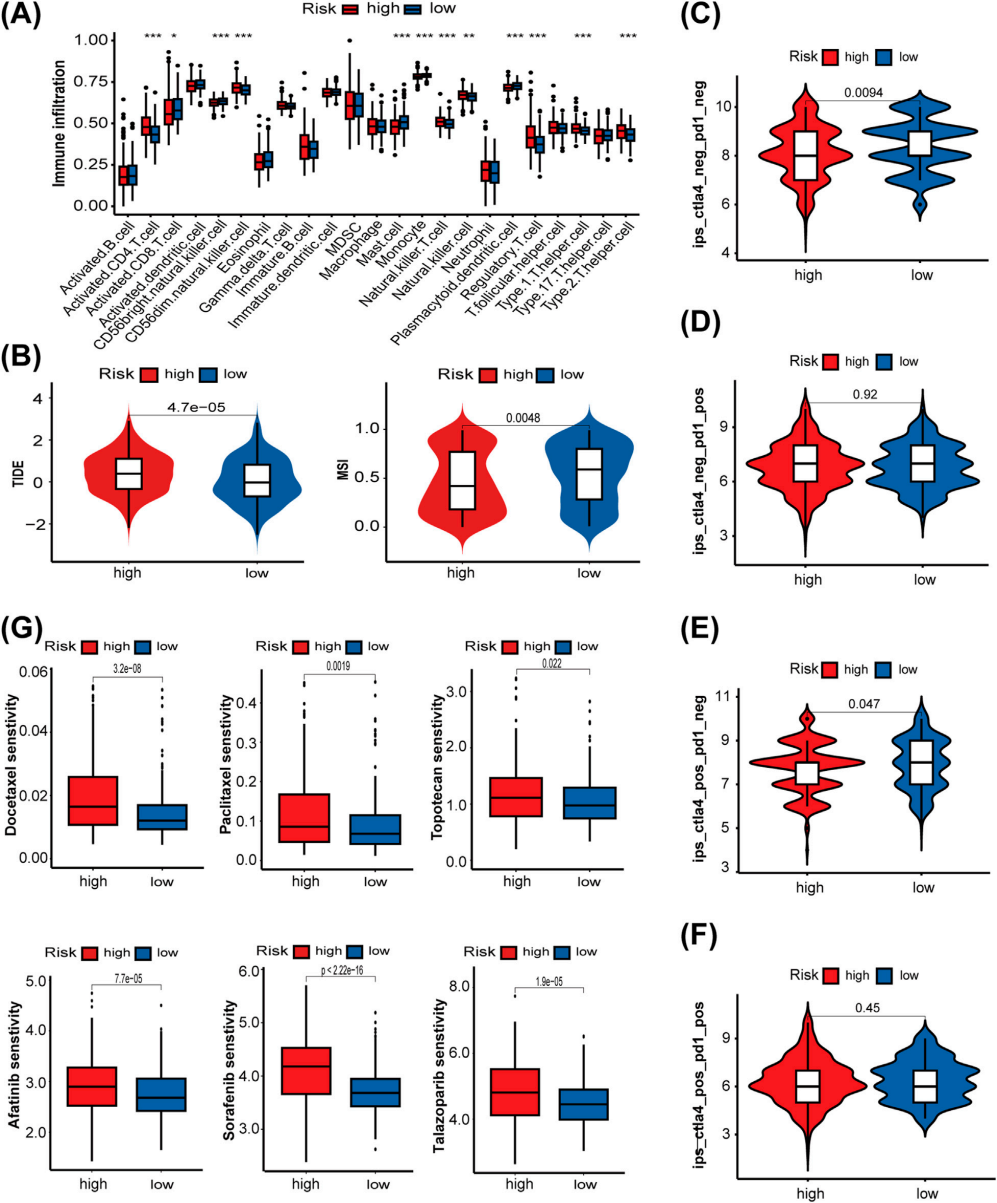
The role of the LMRG/LYG-based signature in predicting the efficacy of antitumor therapy. **(A)** A box plot showing the differences in the infiltration levels of 23 immune cell types between the two risk groups, classified according to the LMRG/LYG-based signature. **(B)** TIDE scores and MSI scores in the different risk groups. **(C–F)** The association between the different risk groups and immunophenoscores. **(G)** Box diagrams showing the sensitivity of the high-risk and low-risk groups for six commonly used drugs.

Next, we used the ssGSEA algorithm to compare immune cell infiltration between the two risk groups. The high-risk group exhibited substantial infiltration of multiple helper T-cells, whereas the low-risk group demonstrated high infiltration of activated CD8^+^ T cells, CD56^bright^ natural killer cells, and plasmacytoid dendritic cells (Figure 7A). These findings reflect a more active immune state in the low-risk group, highlighting clear differences in immune profiles between the two groups. To further confirm the association between the LMRG/LYG-based signature and immune infiltration, immune cell fractions were estimated using TIMER, EPIC, CIBERSORT, and quanTIseq algorithms (Supplementary Figure S4). Then, a series of evaluation metrics was employed to determine whether our risk model was correlated with the response to immunotherapy. The high-risk group had a higher TIDE score along with a lower microsatellite instability (MSI) score than the low-risk cohort, indicative of stronger immune dysfunction and resistance (Figure 7B). In contrast, patients in the low-risk group exhibited higher IPS values, signifying that they had a superior response to immunotherapy (Figures 7C–F). These findings indicated that patients classified as low-risk are more suitable candidates for immunotherapy. To further explore the potential utility of the LMRG/LYG-based signature in guiding individualized UCEC treatment, we investigated the relationship between risk scores and sensitivity to chemotherapeutic drugs and small-molecule inhibitors (Figure 7G). We noted that the low-risk group exhibited greater sensitivity to commonly used chemotherapeutic drugs for UCEC, including docetaxel, paclitaxel, and topotecan, as well as three commonly used small-molecule inhibitors, namely, afatinib, sorafenib, and talazoparib. Other drugs with sensitivity differences between the risk groups are listed in Supplementary Table S10.

### 3.7 The expression level and functional verification of the risk gene *PLAAT1*

**FIGURE 8:**
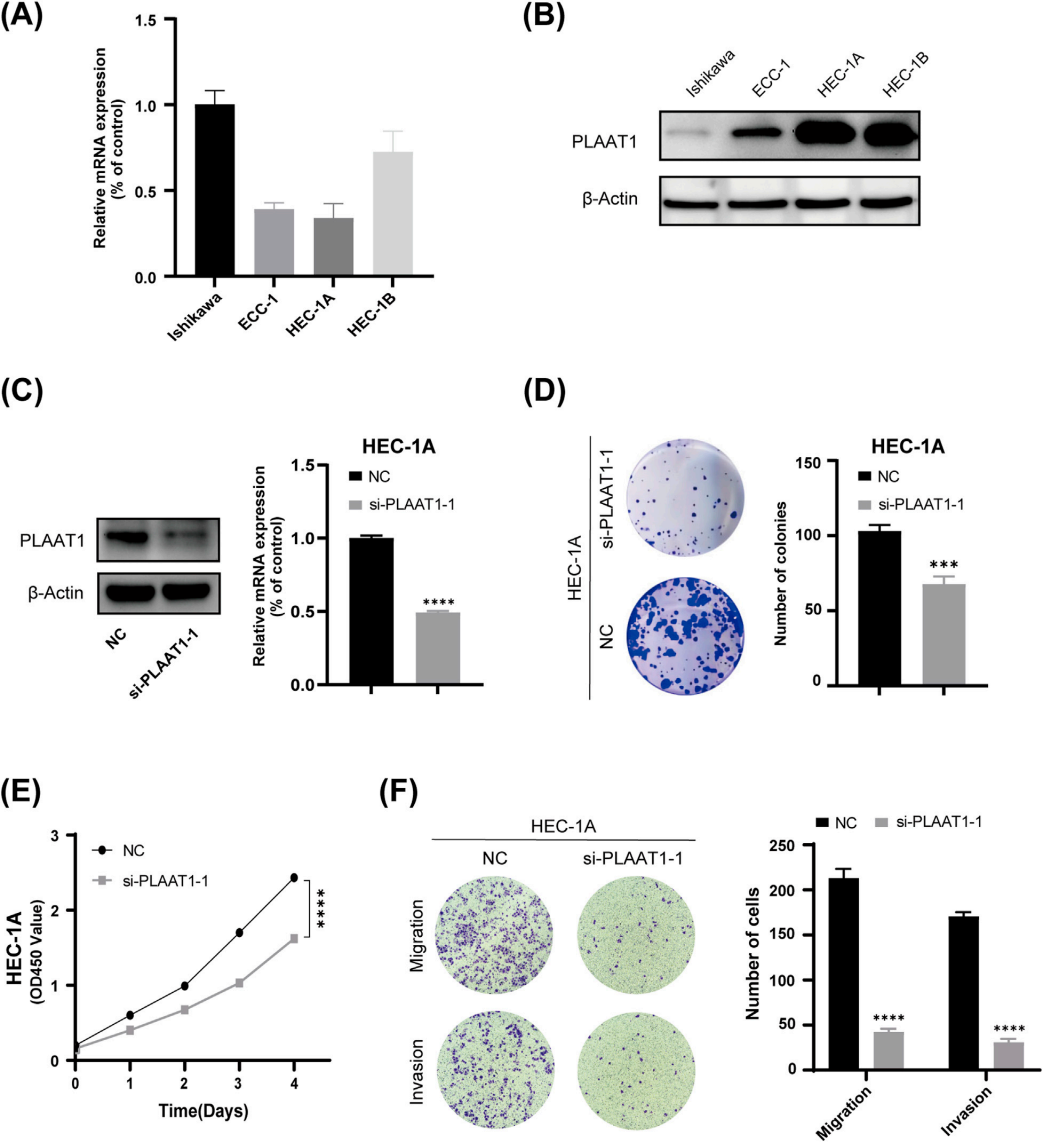
The effect of PLAAT1 on the phenotype of uterine corpus endometrial carcinoma (UCEC). **(A)** The expression level of PLAAT1 in four UCEC cell lines as determined by qRT-PCR assay. **(B)** PLAAT1 protein expression levels in four UCEC cell lines as determined by Western blotting. **(C)** The efficiency of PLAAT1 knockdown after siRNA transfection as determined by qRT-PCR and Western blotting. **(D,E)** Colony formation and proliferation (CCK-8) assays showed that inhibiting the expression of PLAAT1 suppresses the proliferation of UCEC cells. **(F)** A Transwell assay showed that silencing the expression of PLAAT1 inhibited the migration and invasion of HEC-1A cells.

Considering the important role of CD8^+^ T cells in antitumor immune responses, we investigated the association between CD8^+^ T cells and the eight risk genes identified in this study using the TIMER web server (Supplementary Figure S5). Because *PLAAT1* showed the strongest negative correlation with the CD8^+^ T-cell proportion among the eight risk genes, we focused on exploring its biological role in UCEC. First, we found that PLAAT1 was ubiquitously expressed in the four endometrial carcinoma cell lines at both the mRNA and protein levels (Figures 8A,B). Given that HEC-1A cells had the highest PLAAT1 protein level, they were selected for subsequent cytological experiments. To investigate the function of PLAAT1 in endometrial cancer cells, we downregulated its expression in HEC-1A cells through siRNA (*p* < 0.05, Figure 8C). The knockdown of PLAAT1 significantly suppressed cell proliferation, as determined by colony formation and CCK-8 assays (all *p* < 0.05; Figures 8D,E), while also inhibiting cell migration and invasion, as observed in the Transwell assay (all *p* < 0.05; Figure 8F). In conclusion, our data suggested that PLAAT1 may play a carcinogenic role in endometrial cancer. This makes PLAAT1 a promising therapeutic target that merits further study, potentially opening new directions for UCEC treatment.

## 4 Discussion

In recent decades, endometrial cancer has shown a progressive global rise in both incidence and mortality, increasingly affecting younger women. This makes it a major threat to women’s health (Gu et al., 2021). While surgery provides significant survival benefits for early-stage patients, the prognosis for advanced or recurrent metastatic endometrial cancer remains poor. Therefore, exploring new therapeutic targets and developing more effective treatment strategies for these advanced and recurrent cases have become urgent research priorities. Due to the heterogeneity of endometrial cancer, clinical characteristics alone are insufficient for adequately guiding clinical treatment. Incorporating other important factors is thus essential to better guide clinical management and improve the prognosis of patients with endometrial cancer.

Endometrial cancer shows the strongest association with obesity compared to other common malignancies (Crosbie et al., 2010). Obesity, characterized by the abnormal accumulation of body fat, is closely linked to dysregulated lipid metabolism. Reprogramming of lipid metabolism is a prevalent phenomenon in tumor tissues, significantly influencing cancer development and progression. Furthermore, recent work has highlighted the pivotal role of lysosomes in the regulation of lipid homeostasis as well as in cancer progression (Fröhlich and González Montoro, 2023; Machado et al., 2021). While studies have reported the importance of lipid metabolism and lysosomes in UCEC in isolation (Zhang and Han, 2022; Shen et al., 2022), no research to date has combined the two aspects for a comprehensive analysis. Accordingly, in this study, we developed a risk model that incorporates both lysosome- and lipid metabolism-related genes.

First, we used data from TCGA database to perform a consensus clustering analysis and identify lipid metabolism- and lysosome-related genes. After univariate Cox regression and LASSO regression analyses of these genes, eight of them—*LAMP3*, *RNF183*, *EEF1A2*, *PLAAT1*, *ELAPOR1*, *B4GALT1*, *ATP10B*, and *PLA2G10*—were ultimately selected for the prognostic model. Our analysis, which included ROC curves, K-M survival analysis, a nomogram, and calibration plots, consistently demonstrated the model’s favorable predictive performance and practical value. LAMP3 (Liao et al., 2018), RNF183 (Song et al., 2024), ELAPOR1 (Deng et al., 2010), and ATP10B (Wouters et al., 2024) are all localized to the endosome-lysosome compartment, and evidence suggests that they are all involved in the regulation of lipid metabolism, except for ELAPOR1. The genes *EEF1A2* (Jeganathan and Lee, 2007; Tarrant et al., 2016), *B4GALT1* (van den Boogert et al., 2020), *PLA2G10* (Zhang et al., 2023), and *PLAAT1* (Morishita et al., 2021) are also associated with lipid metabolism, although emerging evidence suggests that they may be linked to lysosomal function. While each of these eight risk genes has been reported concerning the progression of many other malignant tumors, their potential roles and associated mechanisms in endometrial cancer have been rarely reported, warranting further research.

Through the TIMER database, we found that among the eight risk genes, PLAAT1 showed the most significant association with CD8^+^ T-cell immune infiltration. Thus, we subsequently focused on its role in endometrial cancer. PLAAT1 belongs to the phospholipase A and acyltransferase (PLAAT) protein family, which exhibits phospholipase A1/A2 and acyltransferase activities (Mardian et al., 2015). Research to date indicates that PLAAT1 is involved in a wide array of biological processes. For instance, Morishita et al. (2021) showed that PLAAT1 is essential for complete lysosomal rupture and degradation in the zebrafish lens. Rahman et al. (2023) found that PLAAT1 exerts phospholipase activity in mouse liver. The absence of PLAAT1 not only weakens the liver’s ability to take up lipids but also inhibits the development of fatty liver in mice fed a high-fat diet by suppressing *de novo* lipogenesis induced by SREBP1c (sterol regulatory element-binding protein 1c) and PPARγ (peroxisome proliferator-activated receptor γ). Further research indicated that SREBP1 is a key hub mediating immune exclusion of hepatocellular carcinoma. Knockout of SREBP1 in liver cancer cells enhances CD8^+^ T cell migration (Dadey et al., 2025). This also partly explains our research findings, namely, that PLAAT1 may mediate the immune exclusion of CD8^+^ T cells through lipid metabolism pathways. Moreover, Lei et al. (2023) proposed PLAAT1 as a valuable prognostic biomarker in lung squamous cell carcinoma. Our research also indicates that PLAAT1 plays an oncogenic role in endometrial cancer, as its knockdown suppressed the proliferative, migratory, and invasive capacity of endometrial cancer cells. These findings support the potential of PLAAT1 as a therapeutic target in endometrial cancer.

In summary, the prognostic model composed of these eight genes was found to be significantly associated with lipid metabolism and lysosomes, and all these genes have been implicated in cancer development. Using the risk score calculated from these eight genes, UCEC patients were subsequently stratified into high- and low-risk groups. Numerous studies have demonstrated that the TMB is strongly associated with the objective response rate (ORR) to ICI therapy, where a high TMB can effectively predict a higher ORR and superior progression-free survival (PFS) (Yarchoan et al., 2017; Johnson et al., 2016; Carbone et al., 2017). The TMB and the LMRG/LYG-based risk score showed a negative correlation in our study, implying that low-risk UCEC patients may exhibit a heightened response rate to ICI therapy. Generally, the TMB can promote the generation of neoantigens, thereby enhancing tumor immunogenicity and ultimately leading to the activation of cytotoxic T lymphocytes (Segal et al., 2008; McGranahan et al., 2016). Furthermore, when we evaluated immune cell infiltration in UCEC samples using ssGSEA, we observed that the low-risk group had a greater abundance of CD8^+^ T cells than the high-risk group, further supporting that this group is more immunogenic. Next, we examined the connection between risk scores and immunotherapy through TIDE, MSI, and IPS analysis. A higher TIDE score is indicative of reduced responsiveness to ICI treatment. The MSI score predicts the degree of microsatellite instability in tumors, with higher scores indicating enhanced sensitivity to ICI therapy (Fu et al., 2020). As anticipated, compared to the high-risk group, the low-risk group exhibited significantly lower TIDE scores and significantly higher MSI scores. The IPS, a robust predictor for the response to anti-PD-1 and anti-CTLA-4 therapies (Charoentong et al., 2017), was also notably higher in the low-risk group, indicative of a greater response rate and increased immunogenicity. Therefore, our risk features provide valuable guidance for ICI treatment in patients with UCEC.

To further validate the clinical utility of the LMRG/LYG-based risk score in predicting treatment benefits for patients, we investigated the association between the risk score and sensitivity to 198 chemotherapeutic drugs and small-molecule inhibitors using oncoPredict. Ultimately, we observed distinct sensitivities to 160 drugs between the low-risk and high-risk groups. Specifically, the high-risk group displayed heightened sensitivity to 20 drugs, with the low-risk group exhibiting elevated sensitivity to the remaining ones. Combined, these data demonstrate that our risk features have the potential for guiding chemotherapy and targeted therapy for UCEC.

While our study offers valuable insights for the evaluation and therapeutic decision-making in UCEC patients, it nevertheless had several limitations. First, the limited sample size of the UCEC cases included in this study may introduce bias into the results. Additionally, the analysis of sensitivity to immunotherapy and chemotherapy was solely based on transcriptomic data, highlighting the need for more prospective experimental validation. Finally, the molecular mechanisms underlying the effects of one of our identified potential targets, PLAAT1, on the proliferation and invasion of endometrial cancer cells require further elucidation.

## Data Availability

Publicly available datasets were analyzed in this study. This data can be found here: The Cancer Genome Atlas (TCGA) dataset (https://www.cancer.gov/ccg/research/genome-sequencing/tcga), Molecular Signatures Database (MSigDB) (http://www.gsea-msigdb.org/), Gene Ontology (GO) (https://geneontology.org/).

## Abbreviations

LMRGs, Lipid metabolism-related genes; LYRGs, Lysosome-related genes; DE-LMRGs, Differentially expressed lipid metabolism-related genes; PRGs, Prognosis-related genes; LM-DEGs, Differential genes among three lipid metabolism subgroups; LMRG-LYGs, Genes related to both lipid metabolism and lysosomes; UCEC, Uterine corpus endometrial carcinoma; TCGA, The cancer genome atlas; MSigDB, Molecular signatures database; GO, Gene ontology; PCA, Principal component analysis; LASSO, Least absolute shrinkage and selection operator; KEGG, Kyoto encyclopedia of genes and genomes; GSVA, Gene set variation analysis; TMB, Tumour mutational burden; TIDE, Tumour immune dysfunction and exclusion; IPS, Immunophenoscore; DEGs, Differentially expressed genes; OS, Overall survival; MSI, Microsatellite instability; ROC, Receiver operating characteristic; K-M, Kaplan-Meier; ICI, Immune checkpoint inhibitor; ORR, Objective response rate; PFS, Progression-free survival; CDF, Cumulative distribution function; IHC, Immunohistochemistry.
