# Supplementary figures and images for: A lipid metabolism and lysosome-based risk signature for prognosis and immune response prediction in uterine corpus endometrial carcinoma
Source: Front Genet. 2025 Sep 8;16:1594682. doi: 10.3389/fgene.2025.1594682 (PMC12450679; doi:10.3389/fgene.2025.1594682)

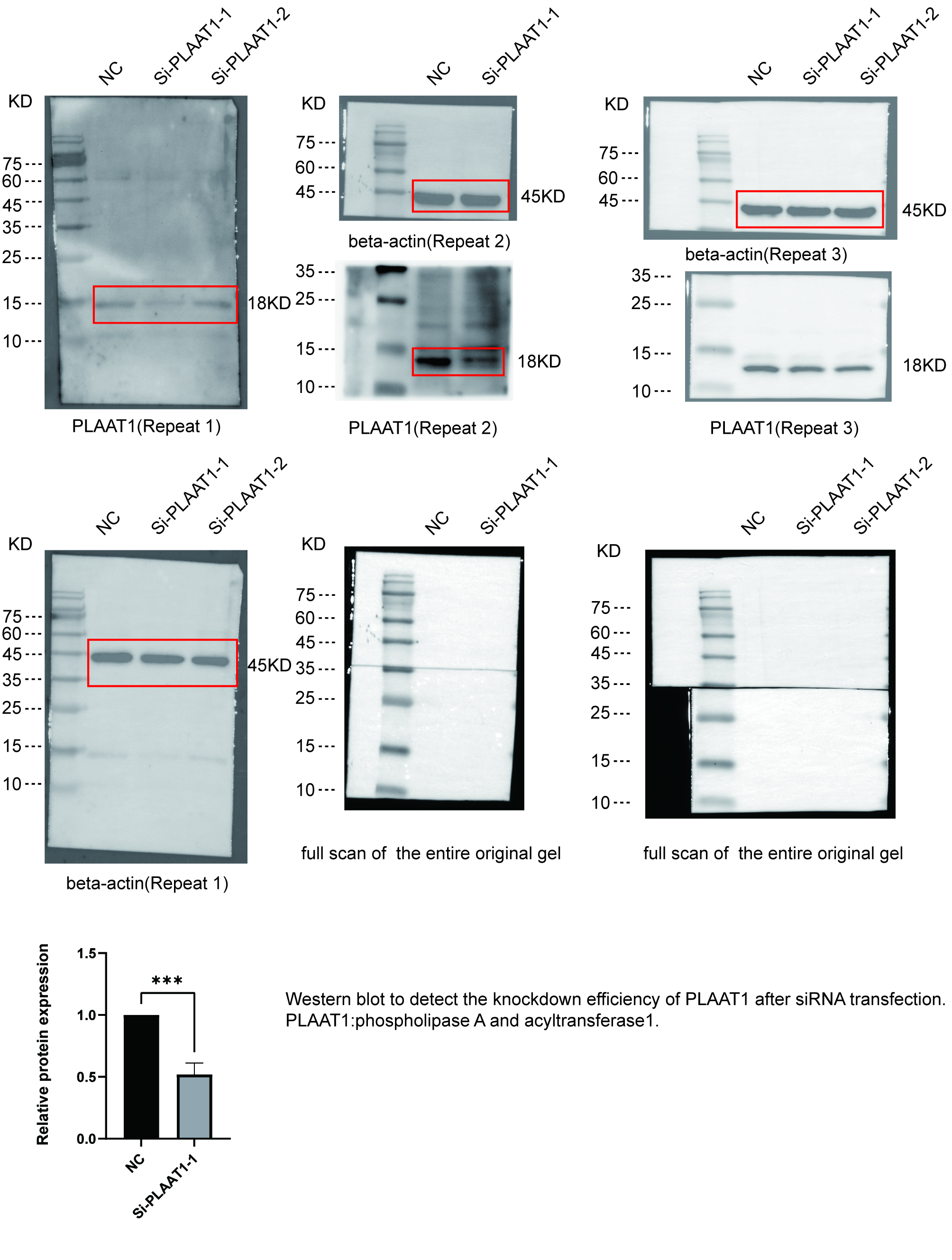

Supplement: Supplementary file 1 [file Image2.tif]

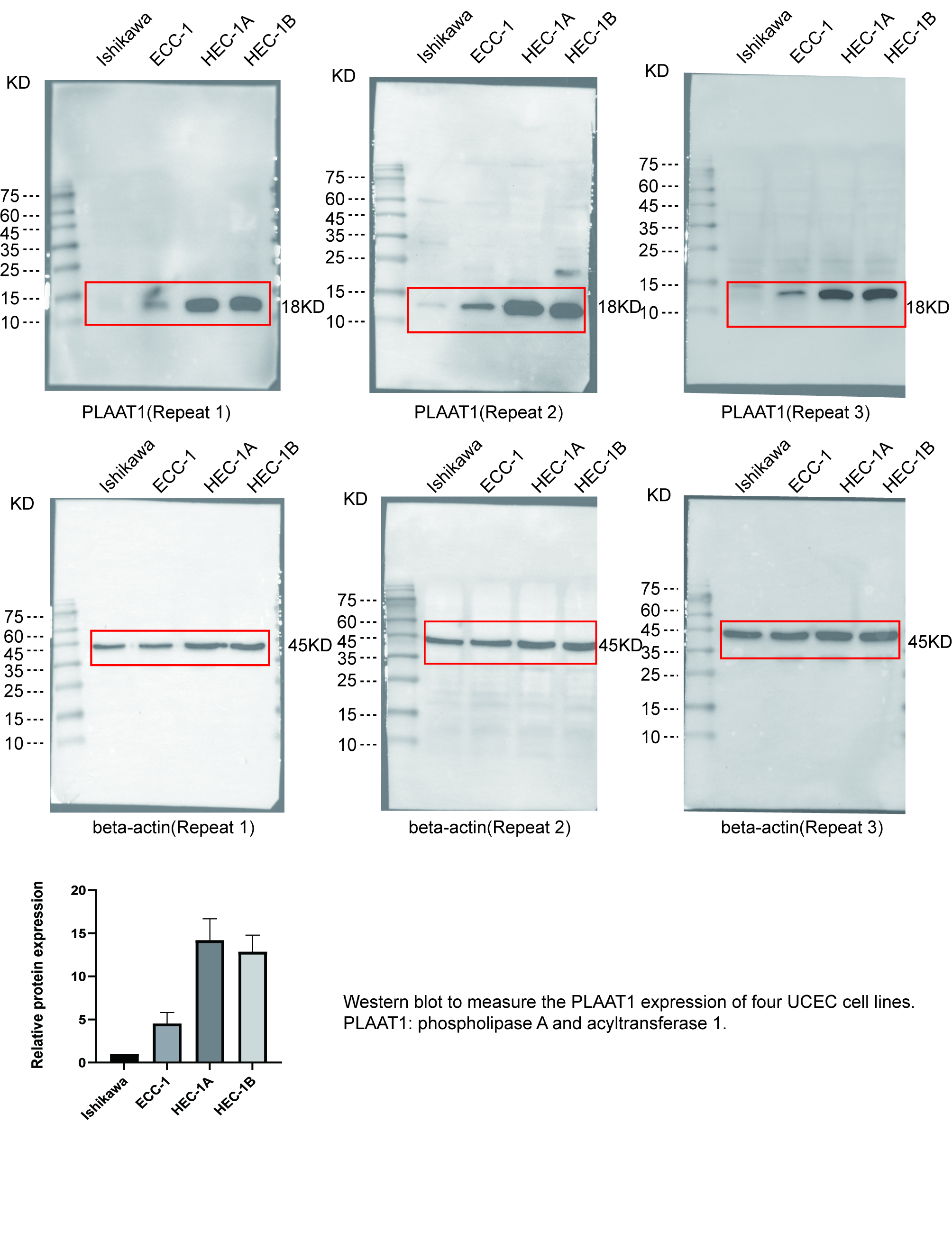

Supplement: Supplementary file 2 [file Image1.tif]
