# Supplementary material for: A lipid metabolism and lysosome-based risk signature for prognosis and immune response prediction in uterine corpus endometrial carcinoma
Source: Front Genet. 2025 Sep 8;16:1594682. doi: 10.3389/fgene.2025.1594682 (PMC12450679; doi:10.3389/fgene.2025.1594682)
Supplement: Supplementary file 3 [file DataSheet1.pdf]

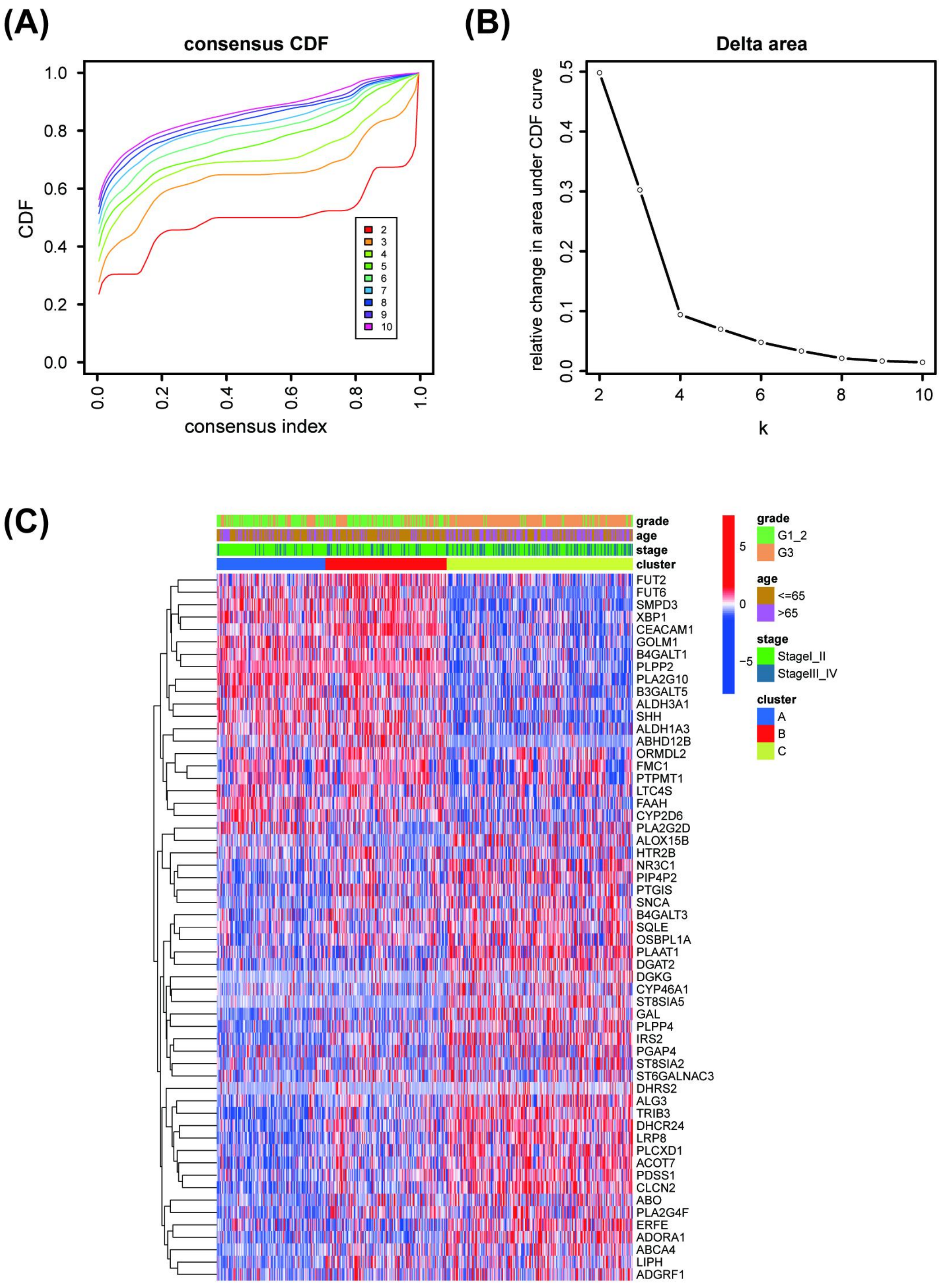

Figure S1. Processes of constructing lipid metabolism clusters. (A) The cumulative distribution function (CDF) plot over the range of k values from 2 to 10 was shown in order to identify the best clustering result. (B) Relative changes in the CDF area for k = 2-10. (C) Expression heatmap of 57 PRGs in three lipid metabolism clusters of the TCGA-UCEC set.

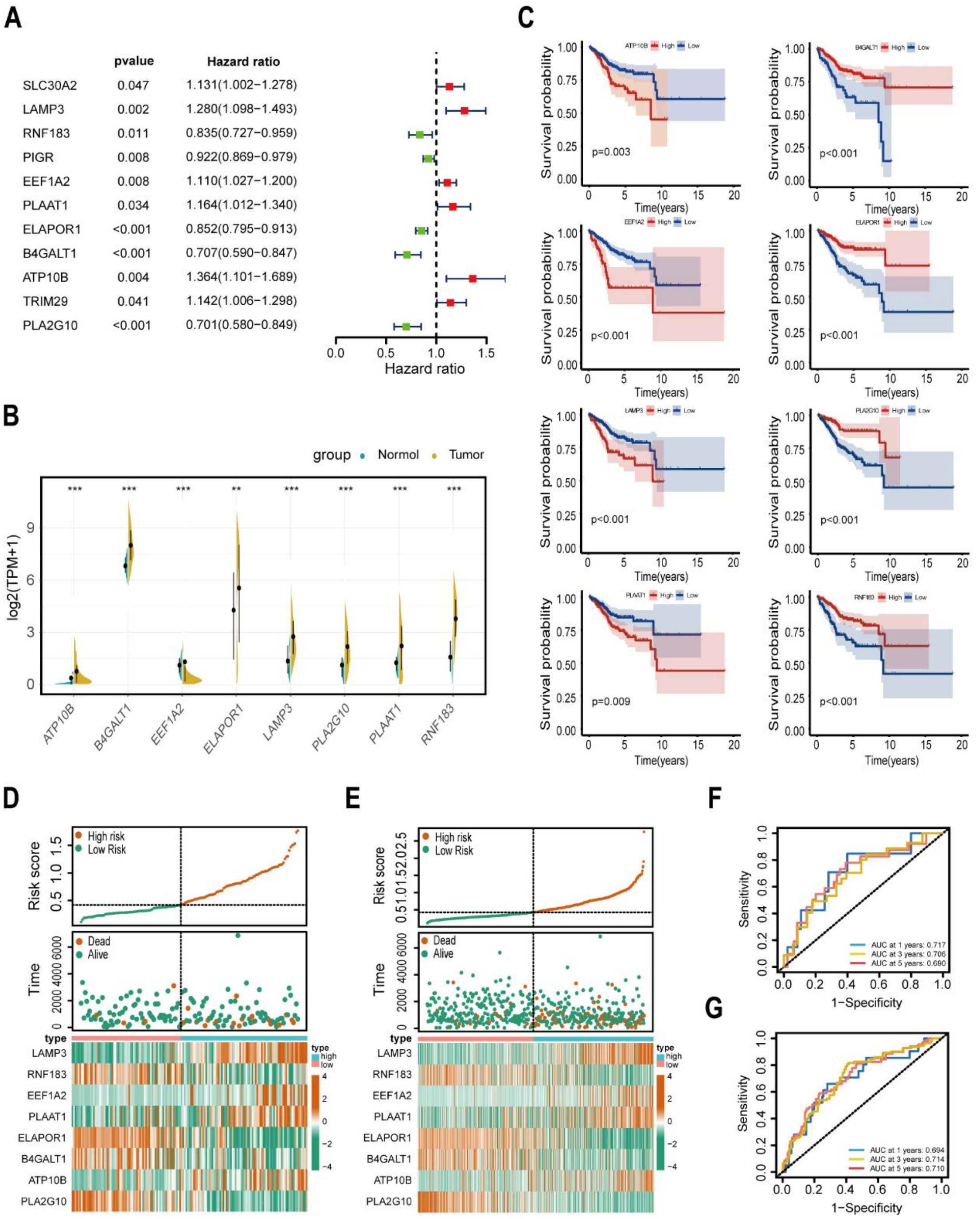

Figure S2. Establishment of prognostic features in UCEC. (A) Univariate Cox analysis of 11 LMRG-LYGs. (B) Violin plot of 8 risk genes expression between normal and tumor. (C) Kaplan-Meier survival curves for 8 risk genes (LAMP3, RNF183, EEF1A2, PLAAT1, ELAPOR1, B4GALT1, ATP10B, and PLA2G10) in the TCGA-UCEC cohort. (D) The evaluation of prognostic characteristics in the testing set. (E) The evaluation of prognostic characteristics in the entire set. (F) ROC curves of predictive performance of the LMRG-LYGs-based signature in the testing set. (G) ROC curves of predictive performance of the LMRG-LYGs-based signature in the entire set.

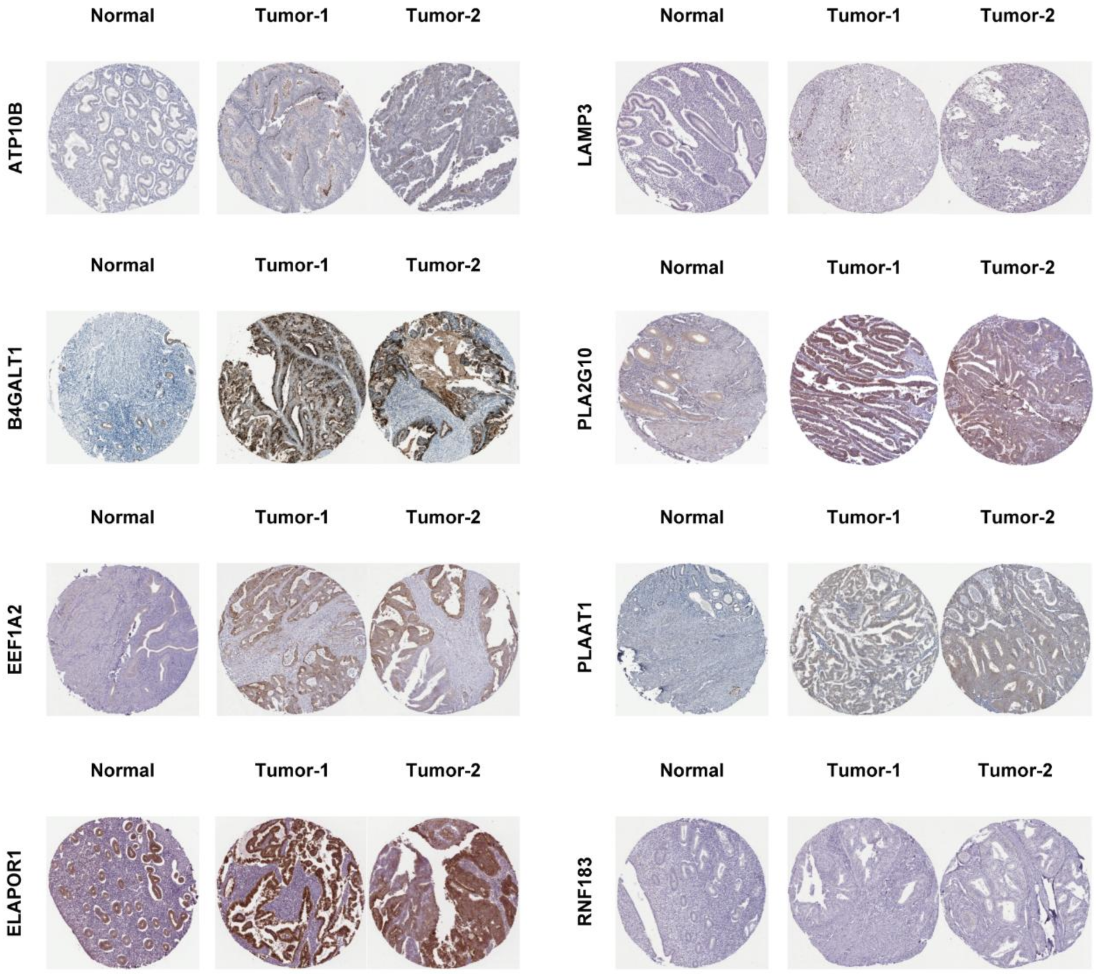

Figure S3. Representative immunohistochemistry (IHC) images of eight risk genes in UCEC in the HPA database.

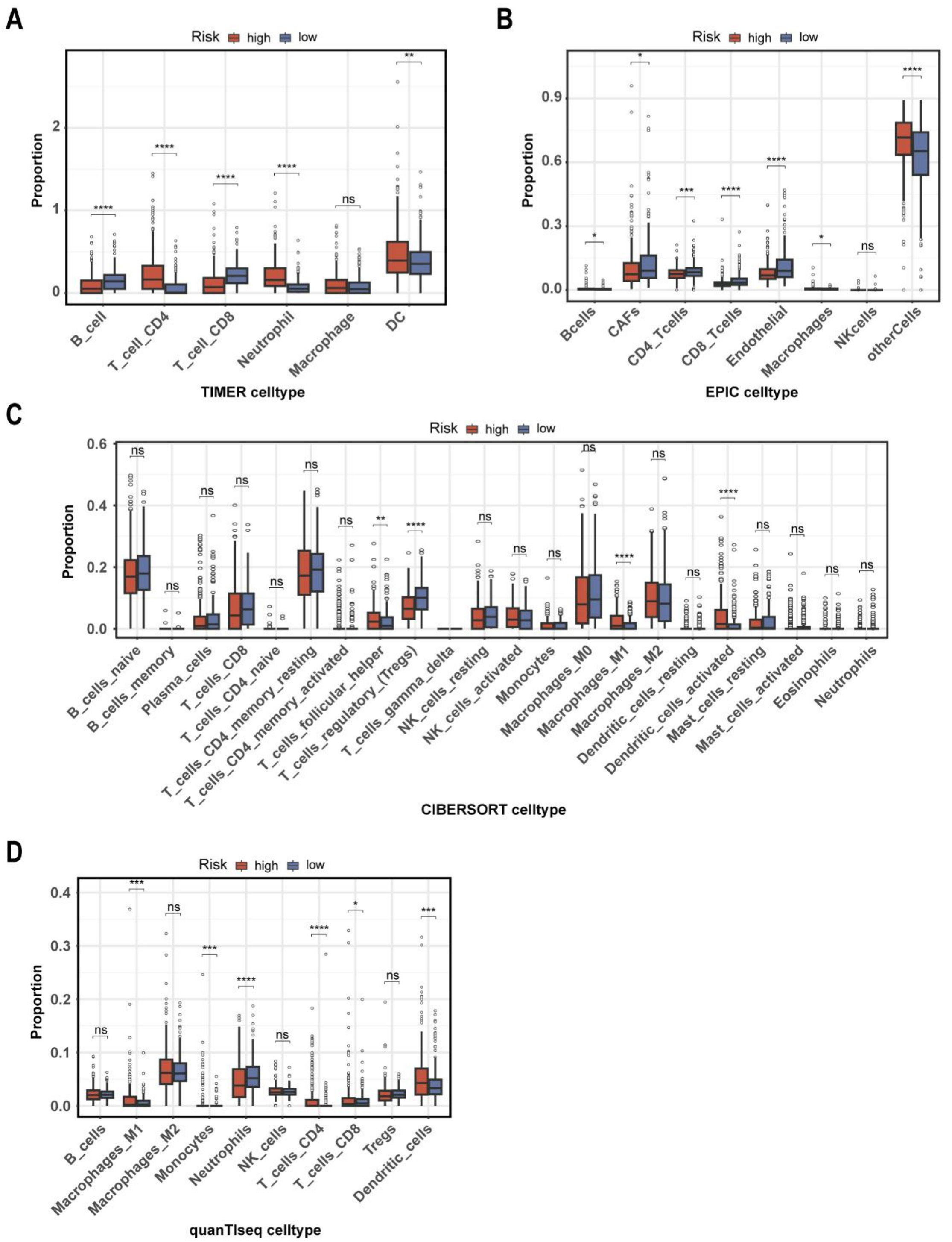

Figure S4. Infiltration of immune cells. Comparison of immune cell infiltration patterns between two risk groups by TIMER (A), EPIC (B), CIBERSORT (C) and quanTIseq algorithm (D), respectively.

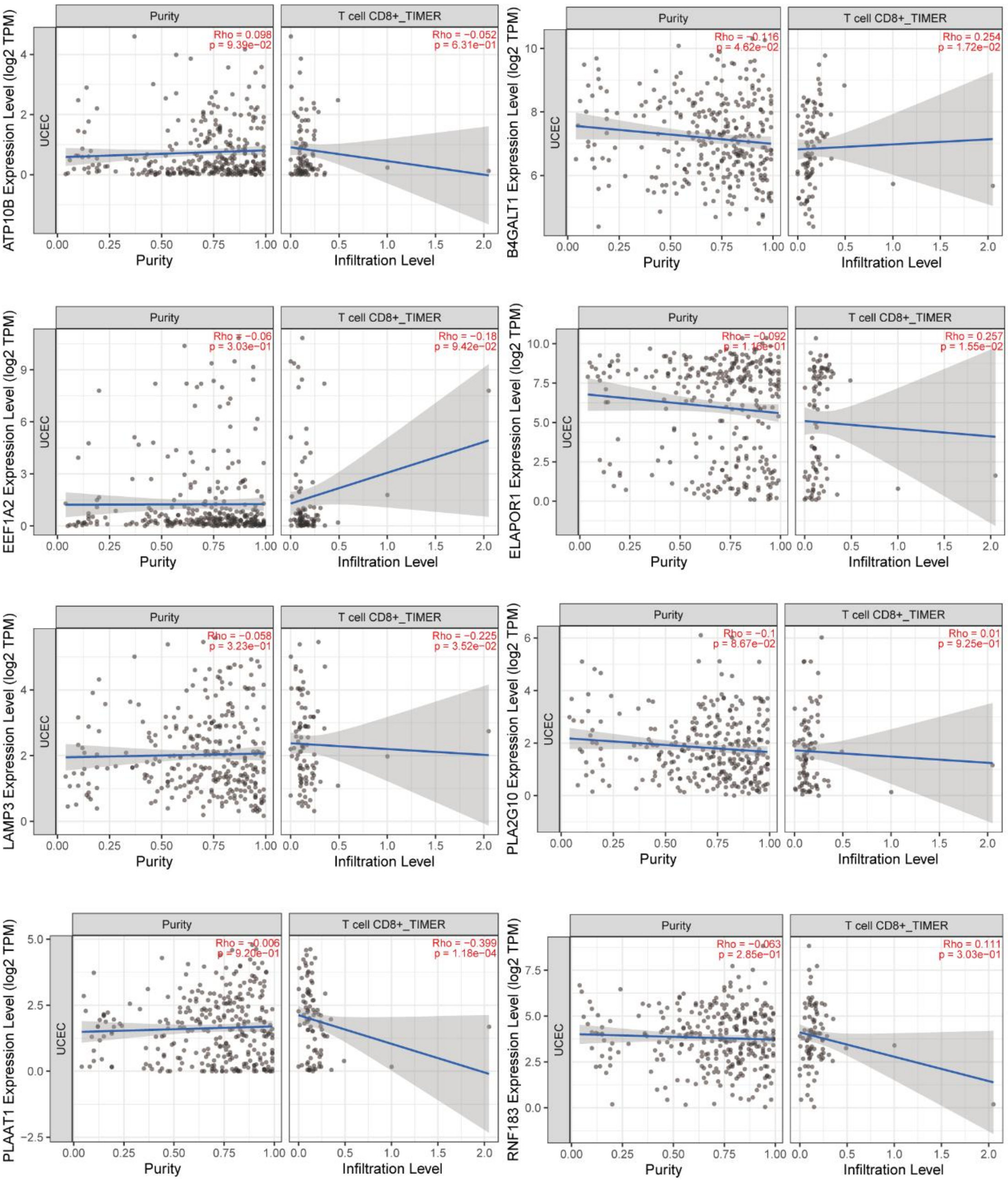

Figure S5. Eight risk genes and CD8+ T cells immune infiltration correlation analyses.
